# Safety and efficacy of nivolumab as a second line therapy in metastatic renal cell carcinoma: a retrospective chart review

**DOI:** 10.3325/cmj.2020.61.326

**Published:** 2020-08

**Authors:** Marija Gamulin, Eric Nham, Deni Rkman, Zrna Antunac, Golubić, Robert Likić

**Affiliations:** 1 Division for Genitourinary Tumors, Department of Oncology, University Hospital Center Zagreb, Zagreb, Croatia; 2 University of Zagreb School of Medicine, Zagreb, Croatia; 3 Division for Clinical Pharmacology and Therapeutics, Department of Internal Medicine, University Hospital Center Zagreb, Zagreb, Croatia; The first two authors contributed equally.

## Abstract

**Aim:**

To assess diseases outcomes and tolerability of real-life second-line nivolumab in a series of metastatic renal cell carcinoma (mRCC) patients.

**Methods:**

This retrospective chart review involved prospectively monitored patients (named patient program) treated with second-line nivolumab for mRCC at the University Hospital Centre Zagreb from February 2016 to March 2018.

**Results:**

The study enrolled 30 patients, 5 of whom (16.7%) had a complete response. The mean ± standard deviation therapeutic response time to nivolumab treatment was 14.07 ± 8.92 months, with a minimum treatment duration of 2 months and a maximum of 24 months. The median duration of therapy was 17 months (mean: 15.8 months; range: 3-24 months), and 50% (n = 15/30) of patients remained alive at the end of follow up. The most common adverse events associated with nivolumab were fatigue (26.67%; n = 8/30), anemia (10.0%; n = 3/30), adrenal insufficiency (6.67%; n = 2/30: G1 = 1, G2 = 1), grade 2 pneumonitis (6.67%; n = 2/30), grade 2 neuropathy (6.67%; n = 2/30), rash (6.67%; n = 2/30: G1 = 1, G2 = 1), and hepatitis (3.33%; n = 1/30).

**Conclusion:**

The present study indicates acceptable patient responses and tolerability of nivolumab in mRCC.

Each year, approximately 337 000 new cases of renal cell carcinoma (RCC) are diagnosed globally, with 143 000 deaths (1). Thirty-three percent of the patients who undergo surgical intervention relapse and additional 33% develop metastases upon initial diagnosis (2,3). A total of 75%-85% of primary kidney malignancies belong to the group of clear cell RCCs, while the remaining histologically diverse tumors are categorized as non-clear cell RCC. Non-clear cell RCCs differ from clear cell RCCs in pathologic and histologic features and clinical presentation (3). Early diagnosis of RCC is crucial as timely surgical intervention can prolong the patient’s life (5-year survival rate of 93%). Unfortunately, a third of the patients already have advanced disease at diagnosis and 10%-20% of them experience a relapse (4-7).

Over the past few years, RCC has been successfully treated with endothelial growth factor receptor tyrosine kinase (VEGF TKI) and mammalian target of rapamycin inhibitors (mTORi) (8). VEGF TKIs are highly effective and can safely be used for several years. However, the median overall survival (22-29 months) suggests that there is still room for improvement (9). Therefore, it is paramount that clinicians have an expert understanding of immunotherapy, as most patients with advanced RCC undergo multiple therapies over the course of their disease (10).

Nivolumab is a PD-1 checkpoint inhibitor that restores the pre-existing antitumor immune response by selectively blocking the interaction between PD-1 receptors on T-cells and PD-1 ligands, PD-L1 and PD-L2, on tumor cells and antigen presenting cells. A 2012 phase I trial demonstrated nivolumab’s antitumor activity and manageable safety profile in metastatic RCC (11). In 2015, nivolumab was approved (CheckMate 025) by Food and Drug Administration (FDA) and the European Medicines Agency (EMA) as a second-line therapy for advanced RCC patients with prior anti-angiogenic therapy, and in 2018 by the EMA as the first-line therapy for patients with intermediate- and poor-risk advanced RCC (12,13).

Since approval, a number of clinical trials have been conducted to further assess the safety, tolerability, and efficacy of nivolumab, or the combination of nivolumab with another antitumor therapeutic agent, in RCC treatment (14-16). We aimed to assess disease outcomes and tolerability of real-life second-line nivolumab in a series of mRCC patients. Our primary aim was to evaluate the dose response with regards to progression-free survival (PFS), overall survival (OS), and time-to-treatment failure (TTF). The secondary aim was to assess the relative safety of nivolumab as a second-line therapy by assessing nivolumab-related adverse events.

## Patients and methods

In February 2019, we retrospectively reviewed the charts of patients with an indication for treatment with nivolumab for mRCC managed between February 2016 and March 2018 at the Clinical Hospital Centre Zagreb. Patients were identified through the Center's electronic health record database, and all patients who received nivolumab were included. Patient data were collected in compliance with all ethical and regulatory standards regarding patient confidentiality.

The study recruited all patients who took part in a named patient program according to the EMA’s medication registration criteria. Heng and MSKCC/Motzer score models for predicting survival were used as inclusion criteria and for stratification of patients into a favorable-risk group, intermediate-risk group, and poor-risk group. Treatment duration was defined as the period from the initial treatment start date to the date of the last treatment cycle. Response to therapy was defined as either complete response (CR), partial response (PR), stable disease (SD), or progressive disease (PD) according to RECIST (17). Patients in whom it was impossible to determine whether they had progression, regression, or stable disease were classified as “mixed response” patients (“modified RECIST”). The PFS was defined as the time in months between the initiation of therapy to progression of disease or death, whereas OS was defined as the time in months from the initiation of therapy to death. Time-to-treatment failure was defined as the interval from the initiation of therapy to its premature discontinuation, which could have ensued due to different reasons, such as cancer progression, adverse events, patient choice, or patient death. The patients were re-assessed at regular scheduled visits at 2, 3, 6, 9, 12, 15, 18, and 24 months.

Nivolumab was administered at a standard dose of 3 mg/kg every two weeks for up to two years. Patients were asked to visit their oncologist every three months for control and consultation. The follow-up visits included laboratory tests, multi-slice computed tomography (MSCT) of the chest, abdomen, and pelvis, and bone status. Patients with musculoskeletal pain underwent a bone scan. The treatment duration ranged from 3 months (shortest) to 24 months (longest).

All relevant data were collected during treatment and were used to determine the baseline characteristics and adverse events observed during nivolumab therapy. Descriptive statistical analysis was performed using the R programming language, v.3.4.0 (The R Foundation for Statistical Computing, Vienna, Austria) to evaluate the differences in patients’ safety and therapeutic response to nivolumab.

## Results

### Patients’ characteristics

The study involved 30 patients (73% men). The mean age at diagnosis was 60.2 ± 9.72 years, ranging from 33 to 78 years (Table 1). There were 70.67% non-smokers (n = 21/30), 23.33% ex-smokers (n = 7/30), and 6.67% smokers (n = 2/30). A total of 3.33% reported alcohol consumption (n = 1/30). The number of metastatic sites before the first-line therapy ranged from 1 to 5 (50.0% to 3.33%) and metastatic sites were found in various tissues and organs, including the lymph nodes (39.62%; n = 21/30), lymph node mediastinum (18.87%; n = 10/30), bones (16.98%; n = 9/30), adrenal glands (7.55%; n = 4/30), and parotid gland and peritoneum (1.89%; n = 1/30 in each).

**Table 1:** Patients’ outcomes, type and severity of adverse events, and duration of nivolumab treatment for metastatic renal cell carcinoma*

N	Sex	Age (years)	Metastases	Nephrectomy	PHD/TNM	Outcomes at 6 months	Treatment duration (months)	Adverse event and severity	First-line therapy	ECOG	Smoking	Alcohol
1	M	62	L, M, bones	Y	CC, pT3aN0M1	DP	7	Fatigue G2, rash G1	sunitinib 50 mg	2	N-ES	N
2	M	58	L, parotid gland	Y	CC, pT2N1M1	CR	24		sunitinib 50 mg	0	Y	Y
3	F	53	bones	Y	Papillary, pT3N2M0	DP	6	Fatigue G2, anemia G2, rash G2, Guillain-Barré syndrome	temsirolimus 25 mg	3	N	N
4	F	73	bones	Y	CC, pT1bN0M1	SD	22+		sunitinib 50 mg	0	N	N
5	M	63	L, M	Y	CC	DP	22+		sunitinib 50 mg	1	N	N
6	F	61	LN, M, pancreas	N	CC	SD	8	Hypothyreosis G2, adrenal insufficiency G2, hepatitis G4	sunitinib 50 mg	1	N	N
7	M	72	bones	Y	CC, T1	Mix	8		sunitinib 50 mg	3	N-ES	N
8	F	71	L, bones	Y	CC, pT3N0M0	DP	9		sunitinib 50 mg	3	N	N
9	M	68	L, adrenal gland, M	Y	CC, pT3aNxM1	SD	20+	Fatigue G2, hypophysitis G2, adrenal insufficiency G1	sunitinib 50 mg	0	N	N
10	M	68	bones, LN	Y	Papillary type2, pT3a	DP	10		temsirolimus 25 mg	2	N-ES	N
11	F	77	bones	Y	CC, pT2aNxM1	DP	10		sunitinib 50 mg	3	N	N
12	F	66	L	Y	CC, pT3bN0M0	DP	8		sunitinib 50 mg	3	N	N
13	M	38	M, bones, L	Y	CC, pT1bN0M0	PR	19+	Fatigue G2, anemia G2, pneumonitis G2	sunitinib 50 mg	1	N	N
14	M	75	L, forearm	Y	CC, M1	SD	19+		pazopanib 800 mg	0	N	N
15	M	67	L	Y	CC, pT3aN0M0	SD	19+		sunitinib 50 mg	0	N	N
16	M	65	L, bones, adrenal gland, kidney	N	CC, M1	DP	4		sunitinib 50 mg	3	N	N
17	M	68	L	Y	CC, pT2bNxM0	SD	9	CVI	sunitinib 50 mg	2	N-ES	N
18	M	54	L, pleura, brain, subcutis	Y	Papillary, pT3apN1M1	DP	5	Anemia G2, fatigue G3	sunitinib 50 mg	3	N-ES	N
19	M	82	LN, M	Y	CC, pT3bNxMx	SD	10	Pneumonitis G2, neuropathy G2, myasthenia syndrome G1	votrient 800 mg	3	N-ES	N
20	M	86	prostatic urethra	Y	CC, N0	DP	7		sunitinib 50 mg	3	N	N
21	F	84	L, M, LN	Y	CC,M1	CR	15+		votrient 800 mg	1	N	N
22	M	65	L	Y	CC	SD	9	Fatigue G1, colitis first G3 subsequently G4	votrient 800 mg	1	N	N
23	M	60	bones, M, peritoenum, L, LN	Y	CC, pT2N0M1	DP	3		sunitinib 50 mg	3	N-ES	N
24	M	59	bones, L,	N	CC	DP	5		sunitinib50 mg	3	N	N
25	M	56	bones	Y	CC, pT2N0Mx	DP	14+		sunitinib 50 mg	0	N	N
26	M	63	L	Y	CC, M1	SD	15+	Fatigue G1, nephritis G2	votrient 800 mg	3	N	N
27	F	81	L	Y	CC, pT2N0M1	DP	16+	Uveitis G2	sunitinib50 mg	0	N	N
28	M	66	LN, adrenal gland	Y	CC, pT3N0M0	CR	20+	Fatigue G2	sunitinib50 mg	0	N	N
29	M	68	M, LN, mesenteri	Y	CC, T3bN2M1	SD	20+		sunitinib 50 mg	0	N	N
30	M	59	LN, M, L, bones, liver	Y	CC, pT2N0Mx	DP	3		sunitinib 50 mg	3	N	N

*L – lungs; M – mediastinal lymph nodes; LN – abdominal lymph nodes; CC – clear cell cancer; SD – stable disease; DP – disease progression; PR – partial response; CR – complete response; MIX – combination PR/SD/DP; Y – yes; N – no; N-ES – no-ex smoker; PHD – pathohistology diagnosis; TNM – tumor-node-metastases.

Before nivolumab therapy, 90% of patients (n = 27/30) had undergone nephrectomy and had received three types of therapy: 50 mg of sunitinib for 4 weeks:2 weeks/2 weeks:1 week (77%; n = 23/30), 800 mg of continuous pazopanib (17%; n = 5/20), and 2 5mg of temsirolimus weekly (6%; n = 2/30). The Eastern Cooperative Oncology Group (ECOG) score at the start of therapy was 0 in 56.67% (n = 17/30), 1 in 36.67% (n = 11/30), and 2 in 6.67% (n = 2/30) of patients (Table 1).

### Therapeutic response

**Figure 1:**
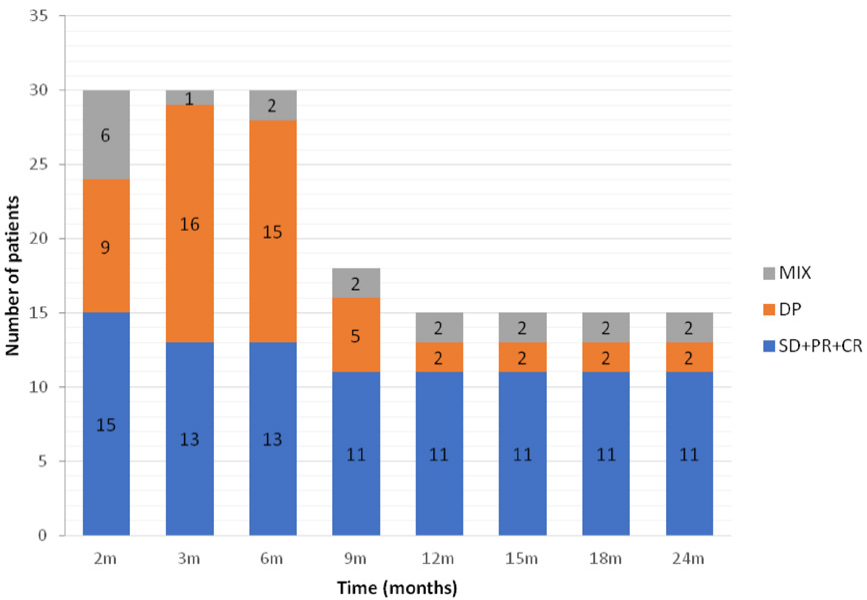
Duration of nivolumab therapy and patient response over the course of treatment. SD – stable disease; DP – disease progression; PR – partial response; CR – complete response; MIX – combination PR/SD/DP.

**Figure 2:**
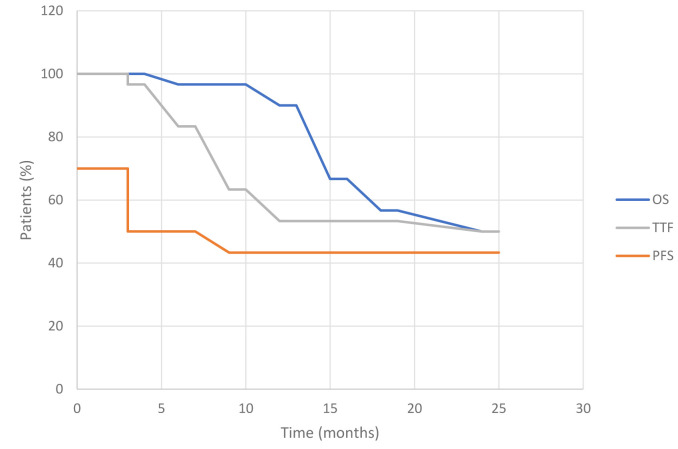
Overall survival (OS), progression free survival (PFS), and time to treatment failure (TTF) curves of 30 patients treated with nivolumab for metastatic renal cell carcinoma.

The mean ± standard deviation response time to nivolumab treatment was 14.07 ± 8.92 months, with a minimum response time of 2 months and a maximum of 24 months. The median duration of therapy was 17 months (mean: 15.8 months; range: 3-24 months). The therapeutic response (stable disease-partial response-complete response, disease progression) is shown in Figure 1 and OS, PFS, and TTF in Figure 2. Patients who were treated with nivolumab for 6 to 12 months, in spite of radiological progression, were ECOG 0-1 and were still alive at the last follow-up visit, while 5 out of 30 (16.7%) had a complete response. At 12 months of follow up, OS was 90% (n = 27/30), PFS 43% (n = 13/30), and TTF 53% (n = 16/30). At 24 months, OS was still not reached as 50% (n = 15/30) of the patients were alive at the last follow-up visit, TTF was 50% (n = 15/30), and PFS remained 43% (n = 13/30) (Figure 2).

### Safety

**Figure 3:**
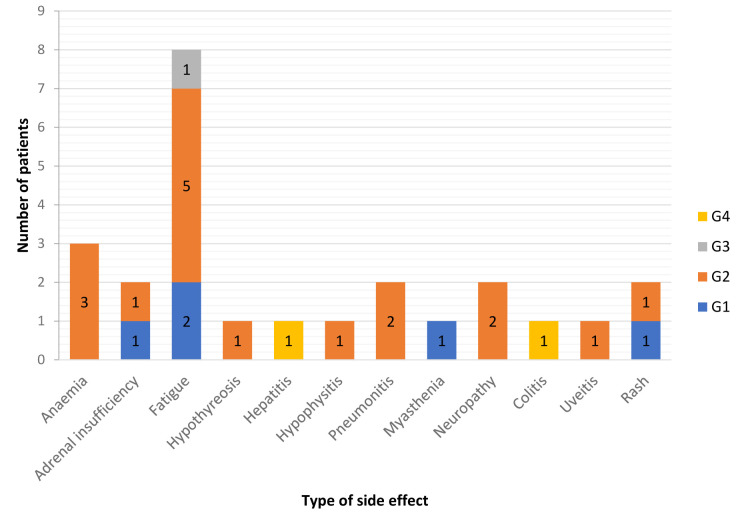
The severity of adverse events that occurred during nivolumab treatment for metastatic renal cell carcinoma. G – grade.

The follow-up period for adverse events was 25 months. The most common adverse event was fatigue (26.67%; n = 8/30), followed by anemia (10.0%; n = 3/30), adrenal insufficiency (6.67%; n = 2/30: G1 = 1, G2 = 1), grade 2 pneumonitis (6.67%; n = 2/30), grade 2 neuropathy (6.67%; n = 2/30), rash (6.67%; n = 2/30: G1 = 1, G2 = 1), and hepatitis (3.33%; n = 1/30). Patients who developed grade 1 and grade 2 adrenal insufficiency, grade 2 nephropathy, grade 2 hypothyroidism, and pneumonitis were successfully managed by the attending physician. After we excluded RCC spread leading to adrenal insufficiency and differentiated an infection from pneumonitis by imaging studies, adverse events were treated with corticosteroids (prednisone 1 to 2 mg/kg per day or methylprednisolone i.v. 1 to 2 mg/kg per day with dose tapering over 4 weeks). In the case of endocrine toxicity, gland specific hormones were added to corticosteroid regimen. Patients with hepatitis G3 or G4 were not administered mycophenolate mofetil, since toxicity started to decrease three days after nivolumab was discontinued. Nivolumab therapy was also stopped in a patient with colitis G4, and toxicity disappeared following corticosteroid treatment (prednisone 2 mg/kg/d) (Table 1 and Figure 3).

## Discussion

Our findings show acceptable patient response and manageable safety profile of nivolumab for RCC, which is consistent with the drug’s recent approvals in this indication by the regulatory agencies. The incidences of immune related adverse events in this study were comparable with those described in the literature (18,19). Furthermore, nivolumab with its mean duration of treatment of 14.07 ± 8.92 months appears to be a good second-line therapy option for patients who also had a long response (>1 year) time to TKI in the first line therapy.

In the last few years advanced RCC has been successfully treated by combinations of immunooncologic therapies. However, the optimal drug sequential therapy for every patient remains to be found. The outcome of mRCC has improved with the advancement of targeted therapies (20). There are seven targeted therapies currently available for clear cell RCC: VEGFR TKIs (sorafenib, sunitinib, pazopanib, and axitinib); VEGF-directed monoclonal antibody bevacizumab (approved in combination with IFN); and mTORi (everolimus and temsirolimus). Although these agents achieved a positive impact, long-term responders are rare (21). High-dose IL-2 is the only approved agent to produce complete durable responses, but hospitalization and intense monitoring are still required (22).

In one of the first trials with nivolumab on 236 patients who received the drug every two weeks (0.1-10 mg/kg), responses were identified in NSCLC (18%), melanoma (28%), and RCC (27%), many of which were durable (10). Since the FDA’s and EMA’s approval of nivolumab as a second-line agent for therapy of advanced RCC, a number of clinical trials and studies have confirmed its effectiveness and safety in that indication (18,19,23,24). ChekMate 025 trial, conducted during more than 5 years in metastatic clear cell RCC patients, found that nivolumab was a better treatment choice than everolimus, with a median OS of 25.8 months vs 19 months (25). Nivolumab also showed improved PFS.

The limitations of this study include a small study sample and potential information and selection bias. Additionally, we did not use cabozantinib, as it is not reimbursed by the Croatian Health Insurance Fund. Despite such potential weaknesses, we believe that our results verify the current direction of investigations into the effectiveness and safety of nivolumab, thus reaffirming this drug’s potential to become the standard treatment for patients with advanced RCC with prior antiangiogenic therapy (12,24). Since immunotherapy in the second-line treatment demonstrated good results in metastatic clear cell RCC patients, a combination of immuno-immuno therapy (nivolumab/ipilimumab) or immuno (pembrolizumab or avelumab) and TKI (axitinib) therapy is increasingly being used as the first-line treatment (26).
